# Contralateral seventh cervical nerve transfer for central spastic arm paralysis: a systematic review and meta-analysis

**DOI:** 10.3389/fneur.2023.1113254

**Published:** 2023-08-17

**Authors:** WenMiao Luo, ZhengCun Yan, Yu Guo, Ji Xu, Heng-Zhu Zhang

**Affiliations:** ^1^Northern Jiangsu People’s Hospital, Yangzhou, China; ^2^Department of Neurosurgery, The Yangzhou School of Clinical Medicine of Dalian Medical University, Yangzhou, China

**Keywords:** meta-analysis, rehabilitation therapy, recovery, stroke, surgery

## Abstract

**Objectives:**

The specific benefits of a contralateral cervical 7 nerve transplant in people with spastic paralysis of the upper extremity caused by cerebral nerve injury are unclear. To evaluate the efficacy and safety of contralateral C7 nerve transfer for central spastic paralysis of the upper extremity, we conducted a comprehensive literature search and meta-analysis.

**Materials and methods:**

PRISMA guidelines were used to search the databases for papers comparing the efficacy of contralateral cervical 7 nerve transfer vs. rehabilitation treatment from January 2010 to August 2022. The finishing indications were expressed using SMD ± mean. A meta-analysis was used to assess the recovery of motor function in the paralyzed upper extremity.

**Results:**

The meta-analysis included three publications. One of the publications offers information about RCTs and non-RCTs. A total of 384 paralyzed patients were included, including 192 who underwent CC7 transfer and 192 who received rehabilitation. Results from all patients were combined and revealed that patients who had CC7 transfer may have regained greater motor function in the Fugl-Meyer score (SMD 3.52, 95% CI = 3.19–3.84, *p* < 0.00001) and had superior improvement in range of motion compared to the rehabilitation group (SMD 2.88, 95% CI = 2.47–3.29, *p* < 0.00001). In addition, the spasticity in the paralyzed upper extremity significantly improved in patients with CC7 transfer (SMD −1.42, 95% CI = −1.60 to −1.25, *p* < 0.00001).

**Conclusion:**

Our findings suggested that a contralateral C7 nerve transfer, which has no additional adverse effects on the healthy upper limb, is a preferable method to restore motor function.

## Introduction

The clinical incidence of cerebral hemorrhage, cerebral infarction, and traumatic brain injury-induced central spastic paralysis of the upper limbs has gone up recently. From 1990 to 2019, the absolute number of stroke incidents worldwide grew by 70% (67%–73%). As a result of falls, both the number and rate of TBI-related hospitalizations rose among those under 75 years old (from 356.9 in 2007 to 454.4 in 2013 per 100,000 population) (1, 2). Limb paralysis brought on by central nervous system damage was a widespread issue that has a significant negative impact on patients, their families, and society (3, 4). Previous therapies including transcranial magnetic stimulation and hyperbaric oxygen therapy aimed to heal the damaged central nervous system, but the outcomes were often subpar over the long run. One of its medical issues is how to better restore the wounded upper limb’s functionality (5).

Brachial plexus nerve injuries were formerly treated using C7 nerve transposition. Gu et al., inventively used it to cure central upper limb spastic paralysis in 2008 based on considerable theory and tests (6, 7). A transpositional anastomosis was used to join the proximal end of the C7 nerve on the side with paralysis to the distal end of the C7 nerve in the healthy upper limb. Innervation of both upper limbs by the uninjured cerebral hemispheres was accomplished after functional rearrangement of the cerebral hemispheres. It offered a fresh approach to treating spastic arm palsy (8–11).

Numerous research had looked at the healing process of contralateral C7 nerve transfer in the management of central upper limb paresis, however it was unclear if this has any particular advantages (12–14). As a result, we performed a meta-analysis of contralateral C7 nerve transfer for central upper limb spastic palsy based on the body of current research to assess the efficacy of this procedure.

This systematic review and meta-analysis were to: (1) compare the recovery of upper limb motor function in patients with central upper limb paresis in the surgery group to that of the rehabilitation group; (2) evaluate the change in upper limb spasticity status in the surgery group following treatment and compare it to that of the rehabilitation group; and (3) evaluate patient safety following CC7 transfer and the negative effects of nerve disconnect. For example, the impact on the contralateral upper limb’s motor and sensory function, the impact on the healthy upper limb’s fine mobility during the course of prolonged monitoring, Agony following nerve disconnect or the possibility of postoperative infection and bleeding, etc.

## Methods

### Standard protocol approvals, registrations, and patient consents

The PROSPERO registry has the research protocol prospectively recorded under Registration. According to the PRISMA statement (Registration No.: CRD42022363569), this systematic review and meta-analysis was conducted, and it was then reported in accordance with the Moose standards. Furthermore, there was no need for ethics in this meta-analysis (15, 16).

### Search strategy

Searches were conducted on electronic databases such PubMed, Human Repository, Embase, Web Science, VIP, CNKI, CBM, and WFSD. Articles only from 2010 to the present that were randomly chosen as controls were chosen. MESH (paralysis and upper extremity) and non-MESH (CC7 nerve, seventh cervical nerve, cervical seventh nerve, C7 nerve, cervical 7 nerve, 7 cervical nerve, etc.) keywords were used for searches (specific search formulae are in the Supplementary material). We conducted a manual search to complete all references that matched the included articles or earlier evaluations in order to fight search incompleteness. The qualifying requirements were last checked on August 10, 2022, to make sure that no additional research matched them.

### Inclusion criteria and exclusion criteria

The following were the inclusion requirements: (1) participants: individuals with cerebral palsy, traumatic brain injury, hemiplegia following, ischemic stroke, hemorrhagic stroke, or both who also exhibited stiffness and weakness in the upper limb contralateral to the brain damage were eligible to participate (patients receiving regular rehabilitation for at least 1 year prior to admission but showing small functional improvement effects in the upper limb). (2) Outcomes: the Fugl-Meyer upper extremity scale, the modified Ashworth scale, and upper extremity range of motion were used to assess motor function and the degree of spasticity in the afflicted limb. (3) Results: the change in UEFM score between the baseline and follow-up was the main result. Modified Ashworth scale (MAS) and range of motion score change from baseline to follow-up were considered secondary outcomes. Spasticity was measured by the MAS score. (4) Published randomized controlled trials.

The exclusion criteria were as follows: (1) absence of source data (2) participants who had previously had bilateral brain malfunction, mental problems, pregnancy, hemorrhagic shock, life-threatening systemic damage, cardiac arrest, chronic sickness, and past severe illness. (3) The literature included case studies, animal experiments, research techniques that did not include CC7, as well as other indicators of study outcomes.

### Study selection

Two impartial examiners (WL. and ZY) in order to find potentially unpublished data, the process is completed by (1) carefully reading the titles and abstracts of all pertinent studies. (2) Manually searching key journals and abstracts of significant annual meetings in the fields of paralysis and CC7 transfer, and (3) contacting experts. The investigators work individually to conduct the majority of the search. Any discrepancies are handled without using the original processes by consulting with the investigators.

### Data extraction

Using a pre-made data extraction form, WL and ZY, two reviewers, independently extracted data. Disputes were settled with a third reviewer (YG). First author, study characteristics (such as year and design), participant characteristics (such as age, country, and sample size), methodological features (such as inclusion criteria, detection, and collecting time), and results were among the data that were extracted (i.e., F-M, MAS, ROM). When it was feasible, assessors contacted the principal author of studies with incomplete data to gather and verify the information. The mean and standard deviation values were extracted using GetData Graph Digitalizer2.24^1^ if the data is graphically or otherwise represented.

### Risk of bias assessment

The quality of the study was reviewed by two impartial assessors. The quality and bias risk of the RCT studies were evaluated using the Cochrane Collaboration’s technique for evaluating risk of bias (17). ROBINS-I assessed the quality and risk of bias evaluation of nRCT publications (18). The two areas of offset risk and applicability were examined. Every category has a unique assessment strategy. We categorize the risk of deviation in each area as low, uncertain, or high based on the strategy utilized to guarantee that each kind of deviation is reduced. In the field of methodology, research whose quality is assessed as low-risk (in all areas) is considered to be of high quality. Any differences will be discussed and resolved by the entire review team.

### Statistical analysis

Based on the raw data, each outcome indicator for patients undergoing CC7 transfer and rehabilitation was evaluated in each randomized controlled and non-randomized research. Consecutive outcomes assessed on the same scale were represented as means and standard deviations and evaluated using standardized mean deviations (SMDS) with 95% confidence intervals (CI) due to potential discrepancies in how they were measured and/or when they were collected. Additionally, in a meta-analytical subgroup study, each upper extremity site’s ROM (elbow, forearm, wrist) and MAS (elbow, forearm, wrist, thumb, fingers 2–4) prognosis was investigated independently. For a pooled examination of the recovery of total motor function in patients who had CC7 transposition, we aggregated all MAS, ROM, and F-M scores. In order to evaluate potential negative effects of CC7 transfer, research data reporting surgical and rehabilitation patients were used to conduct a meta-analysis of adverse events of CC7 in paralyzed patients (risk ratio RR with associated 95% CI). The randomized effect models were selected in accordance with the Cochrane review recommendations if there was significant heterogeneity at *p* < 0.1 or *I*^2^ > 50%; otherwise, fixed-effect models were utilized. This research comprised less than five publications, and no further sensitivity analysis was carried used (19). The software program Review Manager from the Cochrane Collaboration was used for all statistical calculations (RevMan 5.3). And Feng’s et al. (20) nRCT information was eliminated, and outcome indicators were reassessed.

## Results

### Search results

**Figure 1:**
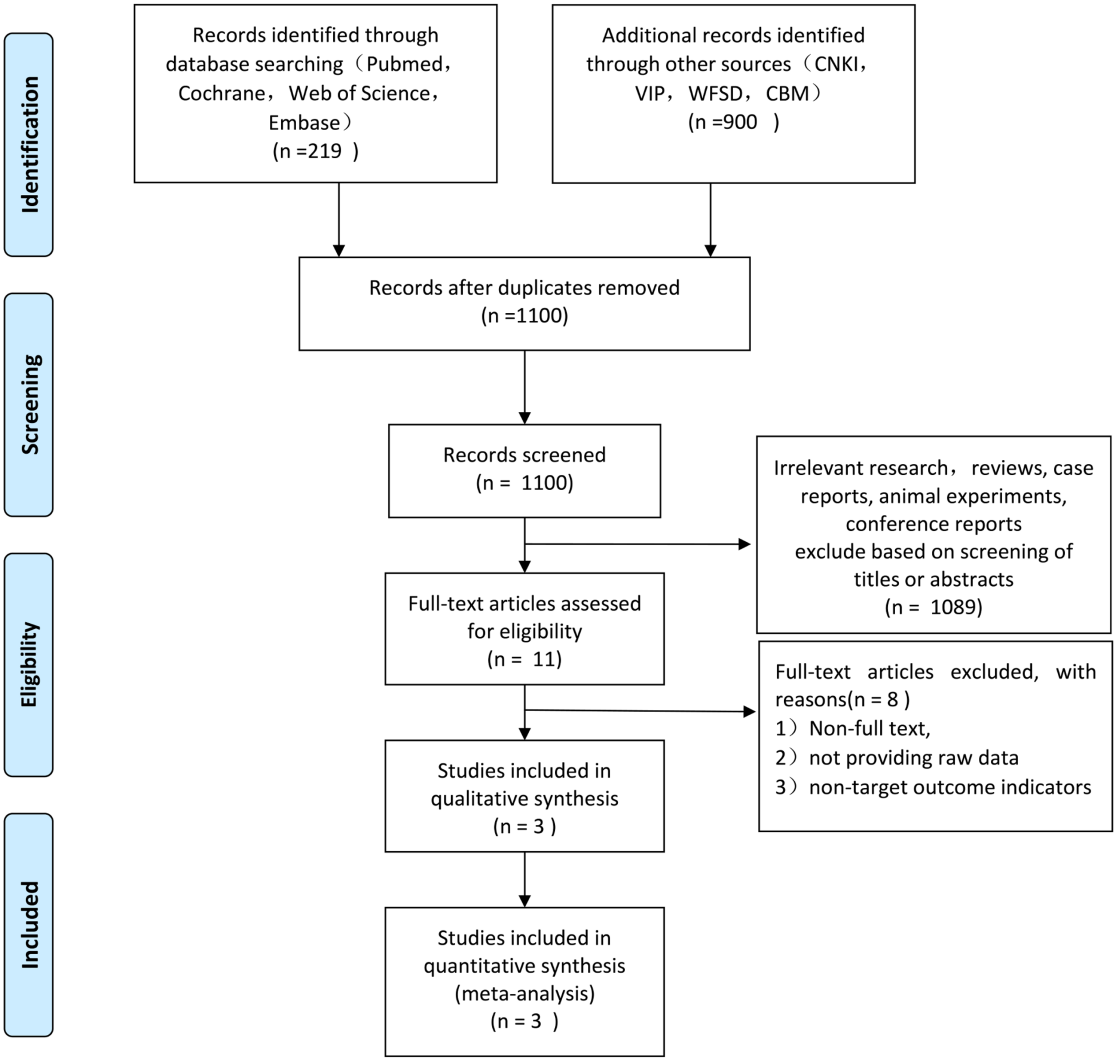
Literature search and screening process.

In accordance with the aforementioned search methodology, 1,119 relevant studies were considered, 19 duplicates were eliminated, and 1,100 studies remained. One thousand eighty nine papers that did not match the inclusion criteria were removed by reviewing the titles and abstracts. Finally, 3 (20–22) English-language articles were accepted after the remaining 8 non-full-text articles were further eliminated in accordance with the exclusion criteria. The flow diagram of literature screening is shown in Figure 1. Included in Feng’s et al. (20) paper were 336 surgical and rehabilitative patients. Seventeen patients in the surgery group and 14 patients in the rehabilitation group met the inclusion criteria for the randomized, controlled trial (refer to the Feng’s Supplementary for specifics).

### Characteristics of included studies

In the meta-analysis, 384 patients from papers published between 2015 and 2022 were included. The age range of the participants in these researches was 4 to 69. The included studies were RCTs and nRCTs (complete data of Feng are from Supplementary Information). These studies mostly described 192 individuals who had complete rehabilitation and 192 patients who received contralateral C7 nerve grafts. The findings showed that following CC7 transfer, the patients’ paralyzed arm had improved motor function and spasticity status. When comparing the two groups at short-term follow-up, it also included the significant negative effects that developed in the surgery group following surgery in comparison to the rehabilitation group. The efficacy and safety of the surgical and rehabilitative groups for treating central upper limb paresis were contrasted in the meta-analysis. Characteristics of included studies were summarized in Table 1.

**Table 1:** Characteristics of the studies included in the meta-analysis.

Study			Feng et al. (20)^a^		Hua et al. (21)	Zheng et al. (22)
Year			2022		2015	2018
Country			China/Korea		China	China
Design			RCT	nRCT	RCT	RCT
Age-y			4–69		21–34	12–45
The cause of paralysis			CNI		CNI	CNI
Time of paralysis-y			≧1		≧1	6–24
Time of follow-up-y			2		2	1
Number of surger-y			17	151	6	18
Number of rehabilitation			13	155	6	18
Outcomes						
The Fugl-Meyer score	Upper-extremity	Surgery	18 ± 4.86	14.82 ± 4.67	17.8 ± 6.37	17.7 ± 5.6
		Rehabilitation	2.08 ± 1.26	2.37 ± 1.82	6 ± 4.2	2.6 ± 2
The modified Ashworth scale	Elbow	Surgery	−0.95 ± 0.66	−0.87 ± 0.57	−1.34 ± 0.89	−0.83 ± 0.62
		Rehabilitation	−0.08 ± 0.28	−0.14 ± 0.57	0.16 ± 0.86	0 ± 0.34
	Forearm	Surgery	−1.12 ± 0.78	−0.95 ± 0.72	−1.34 ± 0.89	−0.89 ± 0.68
		Rehabilitation	−0.23 ± 0.44	−0.20 ± 0.54	0.16 ± 0.86	−0.11 ± 0.47
	Wrist	Surgery	−1.53 ± 0.8	−1.05 ± 0.7	−1.15 ± 0.74	−0.94 ± 0.64
		Rehabilitation	−0.15 ± 0.38	−0.19 ± 0.65	0.02 ± 0.75	−0.17 ± 0.71
	Thumb	Surgery	−1.59 ± 0.62	−1.34 ± 0.62	−1.34 ± 0.45	−1.17 ± 0.71
		Rehabilitation	−0.62 ± 0.77	−0.19 ± 0.47	0.21 ± 0.54	−0.22 ± 0.88
	Fingers 2–5	Surgery	−1.12 ± 0.49	−1.03 ± 0.77	−1.15 ± 0.64	−1 ± 0.69
		Rehabilitation	−0.31 ± 0.85	−0.16 ± 0.56	0.21 ± 0.54	−0.17 ± 0.62
The range of motion	Elbow	surgery	35 ± 15.31	30.03 ± 14.79	25 ± 17.61	24 ± 19
		Rehabilitation	−3.08 ± 6.3	−4.11 ± 6.14	6.67 ± 10.33	0 ± 3
	Forearm	surgery	39.12 ± 15.64	38.11 ± 16.02	66.67 ± 52.03	36 ± 19
		Rehabilitation	−1.54 ± 3.15	−2.32 ± 3.63	5 ± 8.37	1 ± 5
	Wrist	Surgery	45 ± 15.61	37.81 ± 16.08	88.33 ± 19.41	49 ± 21
		Rehabilitation	−2.31 ± 4.39	−2.06 ± 3.95	1.67 ± 4.08	1 ± 5
Adverse events	Bleeding	Surgery	0/17	0/151	NA	0/18
		Rehabilitation	0/13	0/155	NA	0/18
	Infection	Surgery	0/17	0/151	NA	0/18
		Rehabilitation	0/13	0/155	NA	0/18
	Pain	Surgery	7/17	91/151	NA	13/18
		Rehabilitation	2/13	7/155	NA	6/18
	Foreign-body sensation while swallowing	Surgery	3/17	26/151	NA	12/18
		Rehabilitation	0/13	0/155	NA	0/18
	Fatigue	Surgery	5/17	49/151	NA	15/18
		Rehabilitation	0/13	12/155	NA	0/18
	Numbness	Surgery	14/17	145/151	NA	16/18
		Rehabilitation	0/13	0/155	NA	0/18

The literature of Feng offers patients with RCT and non-RCT studies; the Fugl-Meyer score = the Fugl-Meyer upper extremity scale. It was developed to evaluate stroke rehabilitation. The “shoulder and elbow” and “wrist and fingers” domains make up the scale (total score between 0 and 66, with higher scores reflecting better function); the modified Ashworth scale = each joint, including the elbow, forearm, wrist, thumb, and fingers 2–5 fingers, had its level of spasticity evaluated on a scale from 0 to 5. Higher numbers indicate spasticity levels that are greater; the range of motion = the range of motion in the elbow, forearm, and wrist joints of the paralyzed arm. Higher results suggest improved joint range. Joint mobility was assessed between baseline and post-follow up; adverse events = surgical group adverse events during short-term follow-up and rehabilitation group adverse events.

a Data presented as mean.

### Quality assessment

**Figure 2:**
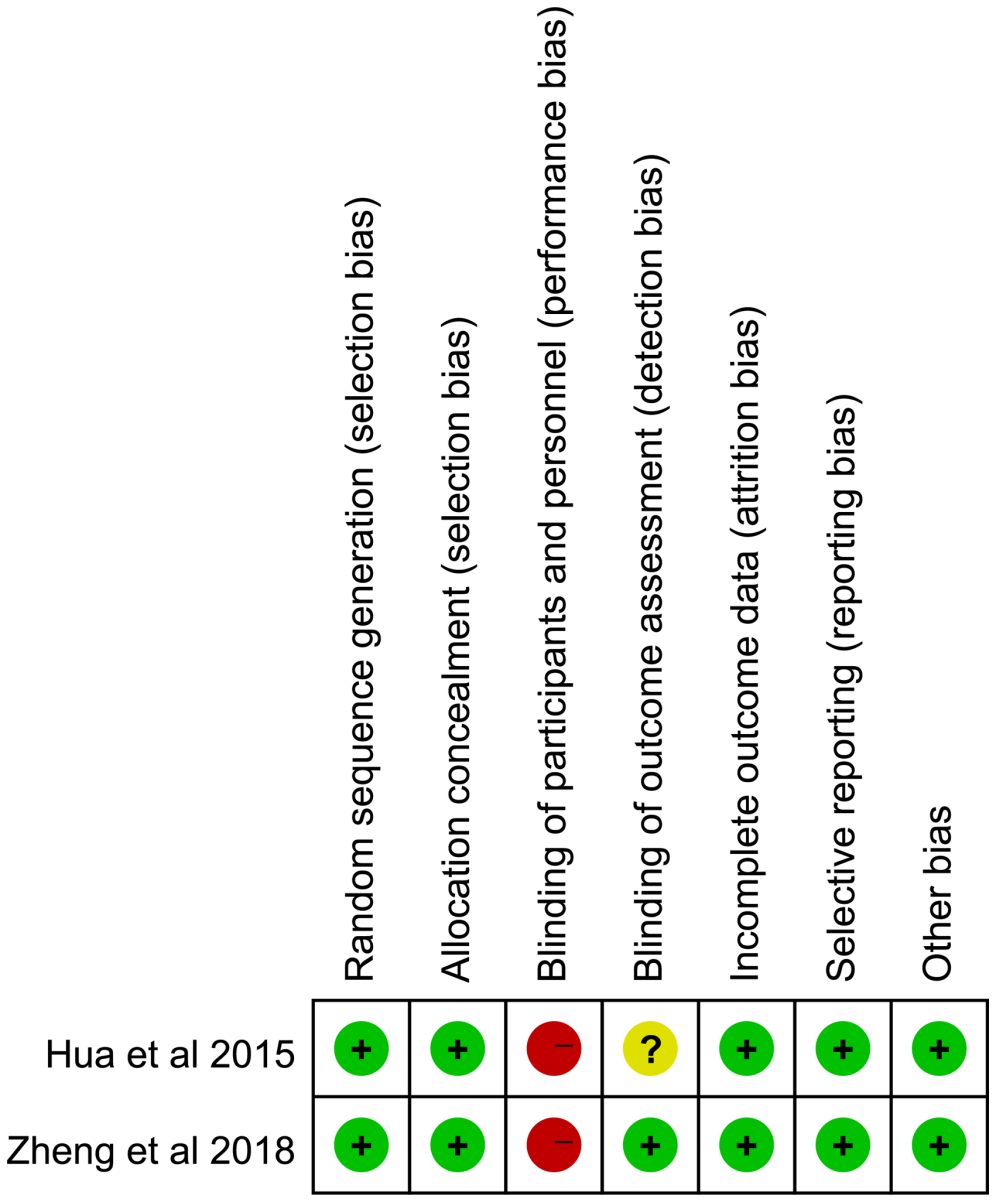
Risk of bias and applicability concerns summary.

In three papers, the risk of deviation and applicability were examined using the Revman5.3 program and the method for measuring bias risk developed by the Cochrane Collaboration. The results of the quality assessment were shown in Figure 2 and Table 2. The three papers’ blinding of participants and personnel involved substantial risk for both parties. The three included English literature articles reported cases of central paralysis with preserved consciousness in patients. Surgical informed consent was obtained from either the patients themselves or their family members. As a result, the assessment of blinding of participants and personnel (performance bias) in the Cochrane Collaboration’s evaluation, as included in the literature, displayed significant deviation. The primary cause of the increased risk was because these papers discussed the procedure’s efficacy, which made it impossible to prevent patients’ giving their informed permission to have surgery. As a result, it was difficult to implement the participants’ blind technique. Overall, the blind technique was where the high risk was mostly focused. The literature included in this research is generally of excellent quality.

**Table 2:** Risk of bias assessment for non-randomized-controlled trials.

Domain	Assessment by outcome
	Feng et al. (20)
Bias due to confounding	Low risk
Bias in selection of participants into the study	Low risk
Bias in classification of interventions	Moderate risk
Bias due to deviations from intended interventions	No information
Bias due to missing data	Low risk
Bias in measurement of outcomes	Low risk
Bias in selection of the reported result	Low risk
Overall	Moderate risk

### Improvement of motor function in patients with central upper limb paralysis

**Figure 3:**
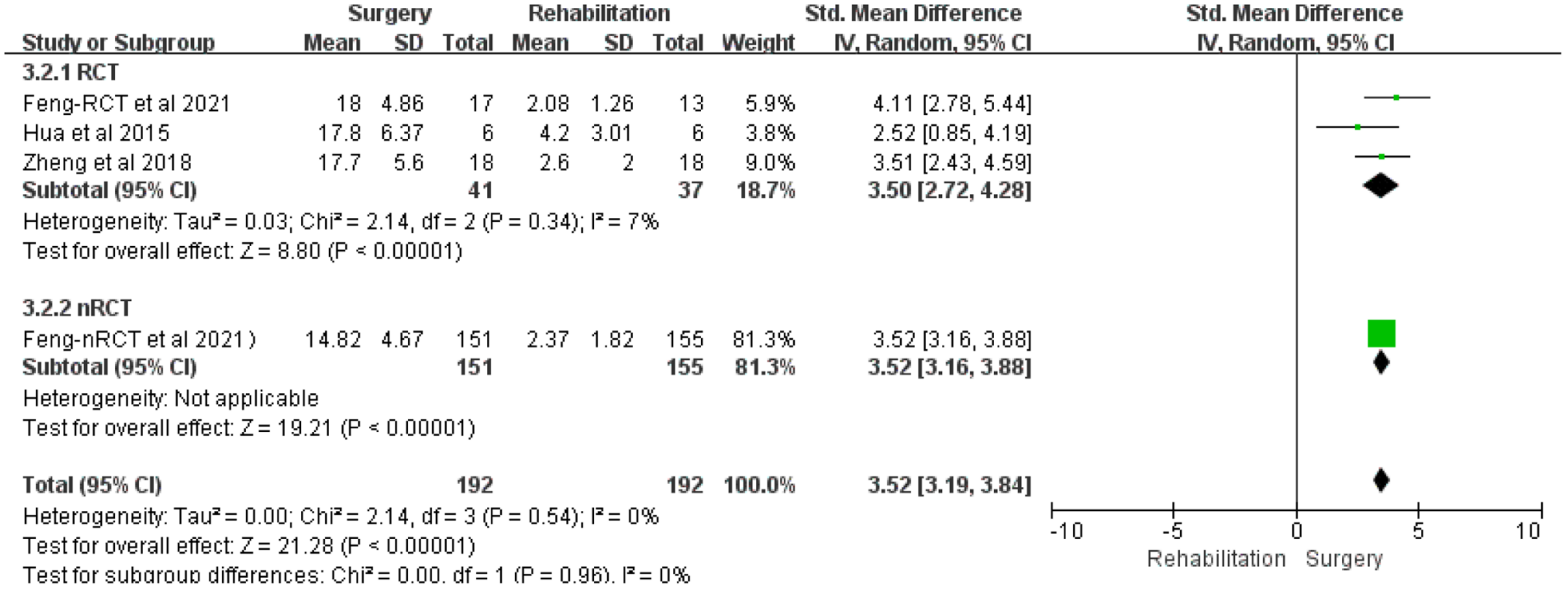
Forest plot: contralateral C7 nerve transfer improves upper extremity motor function in individuals with spastic paralysis of the upper extremities.

**Figure 4:**
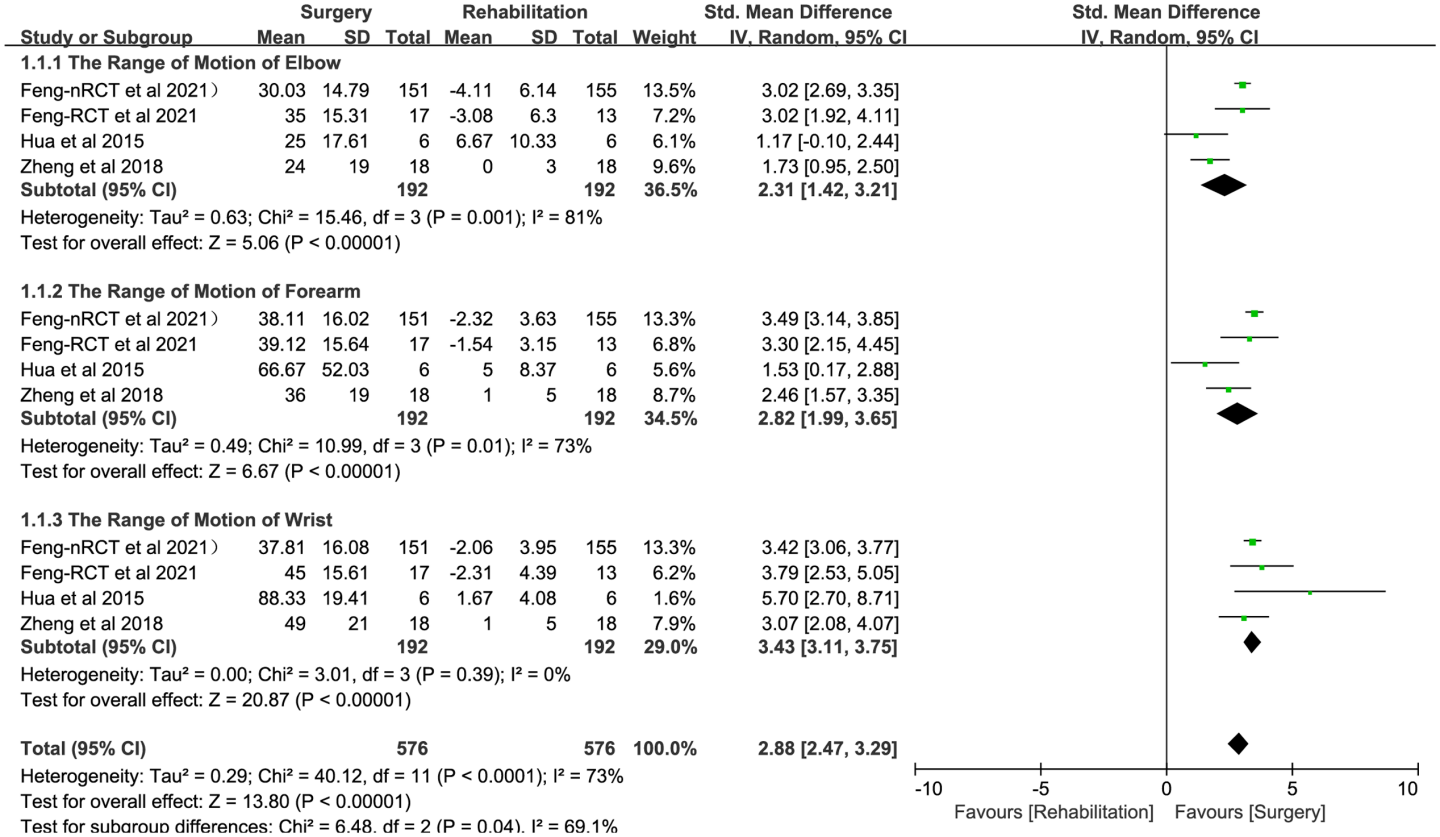
Forest plot: contralateral C7 nerve transfer improves the range of motion of upper extremity joints in individuals with upper extremity spastic paralysis.

All three studies compared the effects of CC7 transfer and rehabilitation (*n* = 192: 192; numbers of RCTs = 41: 37) on the recovery of motor ability in patients with central upper limb paresis and chose them for comparative meta-analysis. The studies looked at changes in upper limb Fugl-Meyer scores and range of motion in the surgical and rehabilitation groups (SMD 3.52, 95% CI = 3.19–3.84, *p* < 0.00001; Figure 3 and Table 3); (SMD 2.88, 95% CI = 2.47–3.29, *p* < 0.00001, *I*^2^ = 73%; Figure 4 and Table 3). The pooled analysis demonstrated that the recovery of upper limb mobility was considerably greater in the paralyzed side of the surgery group than in the rehabilitation group.

**Table 3:** A summary of the overall data or RCT data for several afflicted limb regions.

Subgroup analysis						
Variables	Studies	Effect size (SMD, 95% CI)		*p*-value	*I*^2^%	
		RCTs	RCTs, nRCTs		RCTs	RCTs, nRCTs
F-M scale		3.50 [2.72, 4.28]	3.52 [3.19, 3.84]	<0.0001	7	0
Range of motion						
Elbow	3	1.98 [1.00, 2.96]	2.31 [1.42, 3.21]	<0.0001	63	81
Forearm	3	2.48 [1.59, 3.37]	2.82 [1.99, 3.65]	<0.0001	48	73
Wrist	3	3.62 [2.61, 4.64]	3.43 [3.11, 3.75]	<0.0001	33	0
Modified Ashworth scale						
Elbow	3	−1.61 [−2.13, −1.08]	−1.34 [−1.56, −1.11]	<0.0001	0	0
Forearm	3	−1.35 [−1.85, −0.85]	−1.21 [−1.43, −0.99]	<0.0001	0	0
Wrist	3	−1.46 [−1.98, −0.95]	−1.31 [−1.53, −1.08]	<0.0001	21	0
Thumb	3	−1.38 [−1.90, −0.87]	−1.73 [−2.35, −1.12]	<0.0001	34	65
Fingers 2–5	3	−1.31 [−1.81, −0.81]	−1.29 [−1.52, −1.07]	<0.0001	0	0
Adverse events	3	2.26 [1.20, 4.27]	4.39 [1.04, 18.69]	=0.04	0	87

^*^Data presented as mean or median.

### Improvement of spasticity in patients with central upper limb paralysis

**Figure 5:**
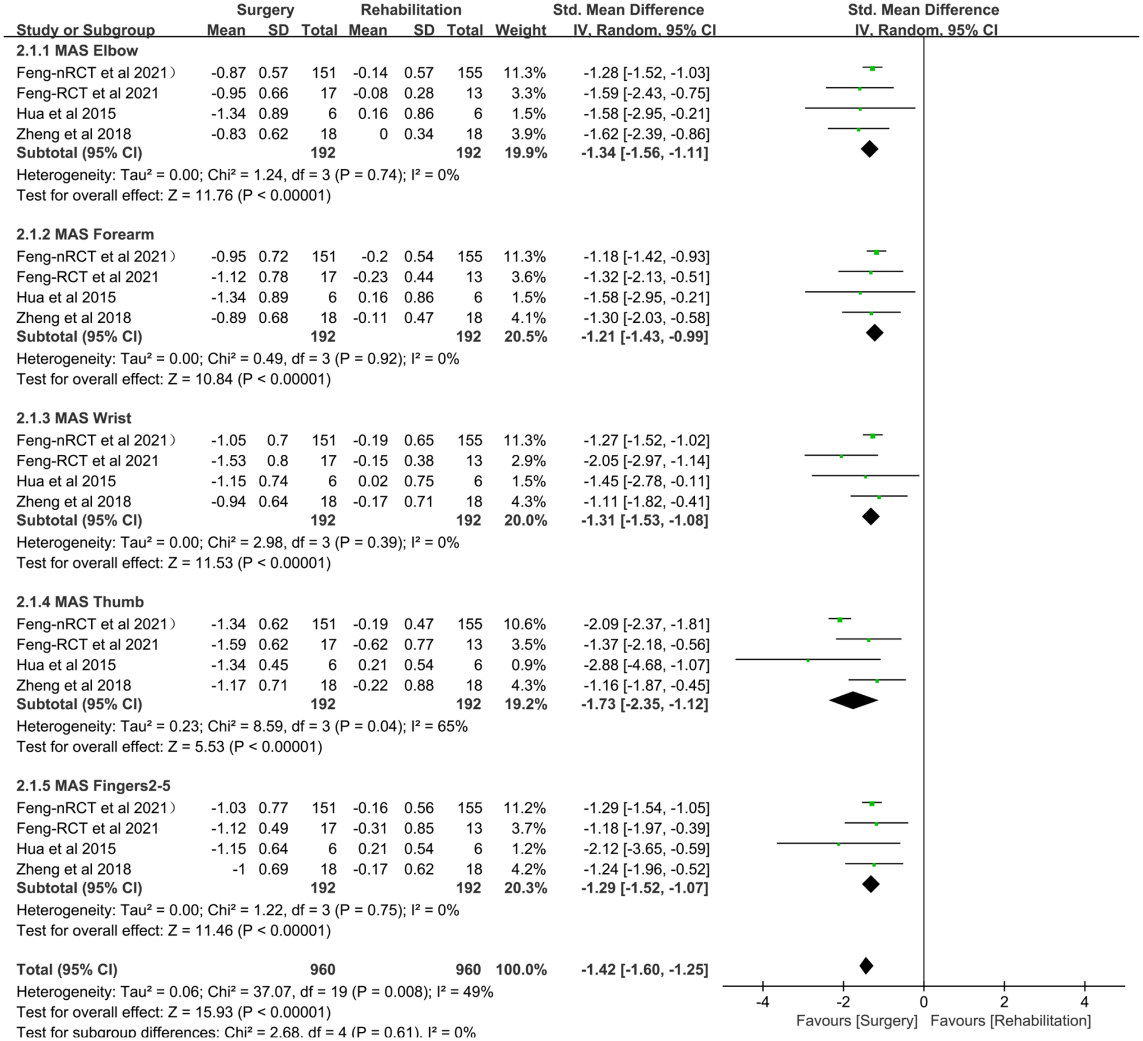
Forest plot: contralateral C7 nerve transfer improves stiffness of the afflicted upper extremity in individuals with spastic paralysis.

All studies assessed the impact of CC7 transfer and rehabilitation on the reduction of upper extremity spasticity (*n* = 192: 192; numbers of RCTs = 41: 37) and were chosen for comparative meta-analysis. The improvement of upper limb spasticity status (MAS) was considerably greater in the surgery group than in the rehabilitation group, according to the pooled analysis (SMD −1.42, 95% CI = −1.60 to −1.25, *p* < 0.00001, *I*^2^ = 49%; Figure 5 and Table 3).

### Adverse events

**Figure 6:**
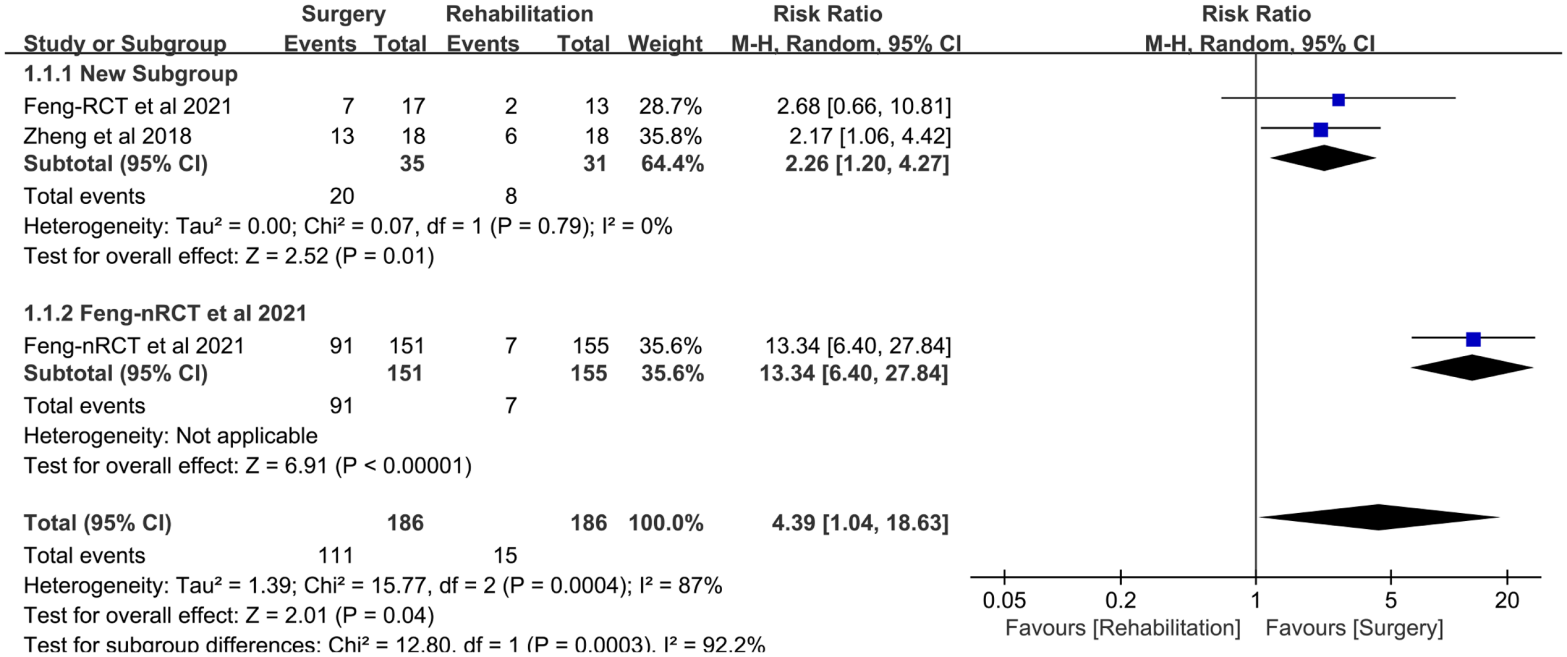
Forest plot: relationship between adverse events and surgery.

CC7 transfer’s adverse events during short-term follow-up were compared with the rehabilitation group (*n* = 186: 186; numbers of RCTs = 35: 31) in two investigations (20, 22). The difference in pain between the two groups was the primary manifestation. Adverse events were more common in the surgical group than in the rehabilitation group but stopped occurring in all patients 6 to 12 months following surgery (RR 4.39, 95% CI = 1.04–18.63, *p* = 0.04, *I*^2^ = 87%; Figure 6 and Table 1).

### Subgroup analysis

Subgroup analyses investigated differences in study outcomes overall and after removing nRCT. Table 2 illustrates the subgroup analysis findings. The findings of the subgroup analysis indicated that there were no significant differences in the RCTs and nRCTs. In addition, the findings of ROM may play a crucial role in the research of heterogeneity.

## Discussion

This meta-analysis assessed the effectiveness and safety of CC7 transfer in the treatment of central upper limb spastic paralysis. The Fugl-Meyer assessment scale and the range of motion of the paralyzed arm indicated a considerable improvement in the motor function of the surgery group, while the modified Ashworth scale revealed a significant reduction in spasticity. Results demonstrated that the upper limb stiffness and motor function were much better in the surgery group. The majority of adverse events in the rehabilitative and surgical groups were pain, and the frequency of adverse events of pain was likely higher in the surgical group (RR 4.39, 95% CI = 1.04–18.63, *p* = 0.04, *I*^2^ = 87%; Figure 6 and Table 1). However, long-term monitoring revealed that all negative effects in patients vanished within 6 to 12 months.

Firstly, Gu et al. suggested cross-transposing the contralateral C7 nerve for central upper limb paralysis. The healthy cerebral hemisphere eventually gained control of both upper limbs after the functional rearrangement of the cerebral hemispheres. The paralysis limbs’ stiffness and motor function were both improved (23, 24). Numerous articles have analyzed the CC7 transfer’s prognosis since its inception, but there has been no consensus on its effectiveness and safety (25, 26). We were the first to employ a more compelling randomized controlled trial in a meta-analysis to investigate the effectiveness and safety of CC7 transfer for central upper limb spastic paralysis. The surgical outcome after CC7 transfer was primarily separated into short-term and long-term outcomes, with the latter being represented in the restoration of motor function in the afflicted limb (27). The literatures that were retrieved from this meta-analysis mostly employed F-M and ROM to assess the patients’ return to motor function, gauge their capacity to move and the range of motion in their afflicted limbs, and calculate an overall index of curative impact. The outcomes of F-M (SMD 3.52, 95% CI = 3.19–3.84, *p* < 0.00001) and ROM (SMD 2.88, 95% CI = 2.47–3.29, *p* < 0.00001) in the surgery groups were significantly higher than those in the rehabilitation groups, further demonstrating that the surgery had a better impact on the motor function of the upper limbs in patients with central paralysis. It is important to note that it was reported that the recovery of range of motion for the patient surgery group was significantly different from that of the rehabilitation group. This may be due to the Hua et al. (21) study’s small sample size, younger patients who were chosen. Manual assessment of upper limb range of motion may be influenced by subjective factors. This, together with the small sample size and the accumulation of risk bias for young patient age, may be a source of heterogeneity in the results.

The alleviation of upper limb spasms was the predominant manifestation of the short-term benefit of CC7 transfer (28). The stretch reflex dysfunction of the central nervous system and muscle spasm were strongly associated. The γ neuron circuit may be reduced and the muscular spasm relieved by successfully cutting the C7 nerve on the side that is afflicted (29). As a result, the amount of alleviation from upper limb spasm may be assessed soon after surgery. The elbow, forearm, wrist, thumb, and fingers 2–5 of the afflicted limb was all thoroughly examined by MAS in the three RCT literatures we chose, and we extracted, analyzed, and assessed the total spasticity alleviation. The CC7 transfer was shown to be effective in reducing the spasticity of the afflicted limb, as evidenced by the suggestion that the MAS in the operation group is considerably lower than that in the recovered group (−1.42, 95% CI = −1.60 to −1.25, *p* < 0.00001).

The primary side effects of CC7 transfer include postoperative complications, exposure to anatomical channel damage, and impacts on the healthy upper limb. The middle trunk of the brachial plexus nerve, the C7 nerve, has been shown to include a significant number of motor and sensory neurons. The brachial plexus nerve’s superior and inferior trunks may carry out the duties of the transected C7 nerve (30). After transection of the C7 nerve in 694 individuals, Li et al. (31), found no reduction in the strength of the contralateral muscles. Numerous studies have shown that most C7 nerve transfer patients suffered symptoms of numbness in the contralateral limb as well as pain from surgical damage, which went away within 2 weeks to 6 months (9, 27). Pain from the damage caused by the procedure’s separation of the nerve and creation of the migration channel is the principal adverse impact described in this article. It is important to note that different anastomoses call for different lengths of the C7 nerve. Some patients needed an alternative anatomic approach, such as an anterior or posterior vertebral approach, or the bridging of additional nerves in order to establish the anastomosis. In two papers, the incidence of adverse events—primarily postoperative pain—in two patient groups was compared. According to the findings, the surgical group had a statistically significant higher rate of adverse events than the rehabilitation group (RR 4.39, 95% CI = 1.04–18.63, *p* = 0.04), and all adverse events vanished after a year of follow-up. Consequently, CC7 transfer is a safe therapy for central upper limb paralysis.

According to reports, the regeneration rate of human nerve anastomosis was around 1 mm/day (32). One month after surgery, Song et al. observed that nerve regenerated axons developed over the anastomosis. One year after the procedure, the distal end of the upper limb was progressively innervated. Guan et al. (33), observed that the average recovery period for motor function following contralateral C7 nerve anastomosis was more than 1 year. After C7 nerve anastomosis, progressively support the damaged limb’s various segments (e.g., elbow, forearm, wrist, etc.). The subgroup study of joint mobility and muscle spasticity recovery in numerous upper extremity locations between the CC7 displacement and therapy groups revealed no significant differences (*p* < 0.00001). In addition, there was no significant impact of 1 year or 2 years follow-up on the recovery of motor function and myospasm in the afflicted limb in the included studies (*p* < 0.00001).

The following are limitations of the presented meta-analysis: (1) only 3 studies were included in the meta-analysis, a rather small amount. Further subgroup and sensitivity analyses were challenging to conduct. Because most of the study’s participants were from East Asia, the results do not accurately reflect their global applicability. (2) The sample size was modest, despite the fact that all of the chosen papers were RCT and nRCT literatures. Individual variations and confounding variables were present. Specifically, in Hua’s et al. (21) paper, the number of patients in the surgery group and the control group was equal at six. Furthermore, the patients were rather young. Due to the limited sample size and large number of confounding variables, it was impossible to do additional research. (3) The damage induced by various surgical methods vary, particularly the exposure and anastomosis of the C7 nerve. For instance, the anterior and posterior spinal approach injured the blood vessels and nerves on the exposure and anastomosis route, resulting in distinct adverse effects. (4) The end index of the study was the score of the score table and the range of mobility of the joint, both of which were susceptible to a substantial subjective effect, and there was a variance in the research findings.

## Conclusion

This is the first meta-analysis assessing the effectiveness of CC7 transfer in the treatment of central upper limb spastic paralysis. The effectiveness and safety of CC7 transfer in the treatment of central upper limb spastic paralysis were evaluated in this meta-analysis. Patients in the procedure group had substantially better F-M and ROM scores when measuring their motor function. At the same time, patients in the surgery group had much less muscular spasm, and there were no major adverse responses. The number of patients presented in this research was limited, the follow-up period was brief, and the impact of individual characteristics on prognosis was not ruled out. Therefore, the efficacy of CC7 transfer warranted more investigation and promotion.

## Author contributions

WL: the concept and layout of the study, data collecting, analysis, and interpretation, as well as paper writing, data collection, analysis, and interpretation. H-ZZ and ZY: the gathering, analyzing, and interpreting of data. YG and JX: revising the manuscript critically for important intellectual content. H-ZZ: the idea and design of the research, as well as a critical revision of the manuscript’s intellectually significant material. All authors contributed to the article and approved the submitted version.

## Funding

This study was funded by the Department of Science and Technology of Yangzhou City (Project No. BRA2019026).

## Conflict of interest

The authors declare that the research was conducted in the absence of any commercial or financial relationships that could be construed as a potential conflict of interest.

## Publisher’s note

All claims expressed in this article are solely those of the authors and do not necessarily represent those of their affiliated organizations, or those of the publisher, the editors and the reviewers. Any product that may be evaluated in this article, or claim that may be made by its manufacturer, is not guaranteed or endorsed by the publisher.

## Footnotes

^1^ https://apps.automeris.io/wpd/

## Abbreviations

CC7: Contralateral C7 nerve
F-M: Fugl-Meyer upper-extremity scale
MAS: Modified Ashworth scale
ROM: Range of motion
SMDs: Standard mean differences
CIs: Confidence intervals
*I* ^2^: I-square
