# Supplementary material for: Contralateral seventh cervical nerve transfer for central spastic arm paralysis: a systematic review and meta-analysis
Source: Front Neurol. 2023 Aug 17;14:1113254. doi: 10.3389/fneur.2023.1113254 (PMC10470831; doi:10.3389/fneur.2023.1113254)
Supplement: Supplementary file 1 [file Presentation_1.pdf]

Search Strategy：only RCT articles in English are included in this article. Therefore, a search strategy for Chinese articles is not provided.

- Pubmed：

((("Paralysis"[Mesh] OR ((((((((((((((Paralyses[Title/Abstract]) OR (Plegia[Title/Abstract])) OR (Plegias[Title/Abstract])) OR (Palsy[Title/Abstract])) OR (Palsies[Title/Abstract])) OR (Hemiplegia[Title/Abstract])) OR (Hemiplegias[Title/Abstract])) OR (Monoplegia[Title/Abstract])) OR (Monoplegias[Title/Abstract])) OR (Hemiplegia, Crossed[Title/Abstract])) OR (Crossed Hemiplegia[Title/Abstract])) OR (Crossed Hemiplegias[Title/Abstract])) OR (Hemiplegias, Crossed[Title/Abstract])) OR (Hemiplegia, Spastic[Title/Abstract])) OR (Hemiplegias, Spastic[Title/Abstract])) OR (Spastic Hemiplegia[Title/Abstract])) OR (Spastic Hemiplegias[Title/Abstract])) AND ("Upper Extremity"[MeSH Terms] OR (Arm[Title/Abstract] OR ("extremities upper"[Title/Abstract] OR "upper extremities"[Title/Abstract] OR "upper limb"[Title/Abstract] OR "limb upper"[Title/Abstract] OR "limbs upper"[Title/Abstract] OR "upper limbs"[Title/Abstract] OR "extremity upper"[Title/Abstract] OR "Arms"[Title/Abstract] OR "upper arm"[Title/Abstract] OR "arm upper"[Title/Abstract] OR "arms upper"[Title/Abstract]) OR "upper arms"[Title/Abstract]))) AND (((((Seventh Cervical Nerve[Title/Abstract]) OR (Cervical Seventh Nerve[Title/Abstract])) OR (C7 Nerve[Title/Abstract])) OR (Cervical 7 Nerve[Title/Abstract])) OR (7 Cervical Nerve[Title/Abstract]))) AND ("randomized controlled trial"[Publication Type] OR "randomized"[Title/Abstract] OR "placebo"[Title/Abstract]) Filters: from 2010/1/1 - 2022/8/7

- **Cochrane**：

Search Name: META C7
Last Saved: 08/08/2022 16:29:43
Comment:

ID Search
#1 MeSH descriptor: [Paralysis] explode all trees
#2 (Paralyses):ti,ab,kw OR (Plegia):ti,ab,kw OR (Plegias):ti,ab,kw OR (Palsy):ti,ab,kw OR (Palsies):ti,ab,kw OR (Hemiplegia):ti,ab,kw OR (Hemiplegias):ti,ab,kw OR (Monoplegia):ti,ab,kw OR (Monoplegias):ti,ab,kw OR (Hemiplegia, Crossed):ti,ab,kw OR (Crossed Hemiplegia):ti,ab,kw OR (Crossed Hemiplegias):ti,ab,kw OR (Hemiplegias, Crossed):ti,ab,kw OR (Hemiplegia, Spastic):ti,ab,kw OR (Hemiplegias, Spastic):ti,ab,kw OR (Spastic Hemiplegia):ti,ab,kw OR (Spastic Hemiplegias):ti,ab,kw
#3 #1 OR #2
#4 MeSH descriptor: [Upper Extremity] explode all trees
#5 (Extremities, Upper):ti,ab,kw OR (Upper Extremities):ti,ab,kw OR (Membrum superius):ti,ab,kw OR (Upper Limb):ti,ab,kw OR (Limb, Upper):ti,ab,kw OR (Limbs, Upper):ti,ab,kw OR (Upper Limbs):ti,ab,kw OR (Extremity, Upper):ti,ab,kw OR

(Arm):ti,ab,kw OR (Arms):ti,ab,kw OR (upper arm):ti,ab,kw OR (arm upper):ti,ab,kw OR (arms upper):ti,ab,kw OR (upper arms):ti,ab,kw

#6 #4 OR #5

#7 (Seventh Cervical Nerve):ti,ab,kw OR (Cervical Seventh Nerve):ti,ab,kw OR (C7 Nerve):ti,ab,kw OR (Cervical 7 Nerve):ti,ab,kw OR (7 Cervical Nerve):ti,ab,kw

#8 #3 AND #6 AND #7

- **Embase:**

No. Query Results Date

#13 #11 AND #12 1 10-Aug-22

#12 'randomized controlled trial':ab,ti OR 'randomized':ab,ti OR 'placebo':ab,ti 1060023 10-Aug-22

#11 #9 AND #10 41 10-Aug-22

#10 [2010-2022]/py 19386452 10-Aug-22

#9 #5 AND #8 44 10-Aug-22

#8 #6 AND #7 25800 10-Aug-22

#7 #3 OR #4 629555 10-Aug-22

#6 #1 OR #2 386106 10-Aug-22

#5 'seventh cervical nerve':ab,ti OR 'cervical seventh nerve':ab,ti OR 'c7 nerve':ab,ti OR 'cervical 7 nerve':ab,ti OR '7 cervical nerve':ab,ti 268 10-Aug-22

#4 'extremities, upper':ab,ti OR 'upper extremities':ab,ti OR 'membrum superius':ab,ti OR 'upper extremity':ab,ti OR 'limb, upper':ab,ti OR 'limbs, upper':ab,ti OR 'upper limbs':ab,ti OR 'extremity, upper':ab,ti OR 'arm':ab,ti OR 'arms':ab,ti OR 'upper arm':ab,ti OR 'arm upper':ab,ti OR 'arms upper':ab,ti OR 'upper arms':ab,ti 382809 10-Aug-22

#3 'upper limb'/exp 354219 10-Aug-22

#2 'paralyses':ab,ti OR 'plegia':ab,ti OR 'plegias':ab,ti OR 'palsy':ab,ti OR 'palsies':ab,ti OR 'hemiplegia':ab,ti OR 'hemiplegias':ab,ti OR 'monoplegia':ab,ti OR 'monoplegias':ab,ti OR 'hemiplegia, crossed':ab,ti OR 'crossed hemiplegia':ab,ti OR 'crossed hemiplegias':ab,ti OR 'hemiplegias, crossed':ab,ti OR 'hemiplegia, spastic':ab,ti OR 'hemiplegias, spastic':ab,ti OR 'spastic hemiplegia':ab,ti OR 'spastic hemiplegias':ab,ti 98451 10-Aug-22

#1 'paralysis'/exp 370017 10-Aug-22

- **Web of Science:**

1: TS=(Paralysis OR Paralyses OR Plegia OR Plegias OR Palsy OR Palsies OR Hemiplegia OR Hemiplegias OR Monoplegia OR Monoplegias OR Hemiplegia, Crossed OR Crossed Hemiplegia OR Crossed Hemiplegias OR Hemiplegias, Crossed OR Hemiplegia, Spastic OR Hemiplegias, Spastic OR Spastic Hemiplegia OR Spastic Hemiplegias)

2: TS=(Upper Extremity OR Extremities, Upper OR Upper Extremities OR Membrum superius OR Upper Limb OR Limb, Upper OR Limbs, Upper OR Upper Limbs OR Extremity, Upper OR Arm OR Arms OR upper arm OR arm upper OR

arms upper OR upper arms)

3: TS=(Seventh Cervical Nerve OR Cervical Seventh Nerve OR C7 Nerve OR Cervical 7 Nerve OR 7 Cervical Nerve)

4: #1 AND #2 AND #3

5: TS=（randomized controlled trial OR randomized OR placebo

6: #1 AND #2 AND #3 AND #5
