# Supplementary material for: Contralateral seventh cervical nerve transfer for central spastic arm paralysis: a systematic review and meta-analysis
Source: Front Neurol. 2023 Aug 17;14:1113254. doi: 10.3389/fneur.2023.1113254 (PMC10470831; doi:10.3389/fneur.2023.1113254)
Supplement: Supplementary file 2 [file Presentation_2.pdf]

Supplementary Appendix

Reconstruction of paralyzed arm function in patients with hemiplegia through contralateral seventh cervical nerve cross transfer: a multicenter study and real-world practice guidance

Table of Contents

Table S1. Changes of primary and secondary outcomes in unmatched cohorts (N=425) 2

Table S2. Patient reported outcomes in the matched cohort. 5

Table S3. Longitudinal data at baseline and time points of each follow-up in the surgery population 6

Table S4. Extended data for changes on muscle strength in the surgery group. 8

Table S5. Adverse events 9

Table S6. Sensitivity analysis on primary outcome. 10

Figure S1. Subgroup analyses of the primary outcome in the surgery group. 11

Figure S2. Patient report outcomes on the reason of why patients in the control group choose rehabilitation over surgery. 12

Figure S3. Extended data for adverse events in muscle strength of the surgery group. 13

Figure S4. Longitudinal data of MAS score in the surgery group. 14

Supporting information 1. Description of the treatments in the surgery and rehabilitation group. 15

Table S1. Changes of primary and secondary outcomes in unmatched cohorts (N=425)

Outcomes	Mean (SD)		Mean (95%CI)	P-value
	Surgery	Rehabilitation	Adjusted mean difference	
**Primary outcome**				-
**Number of patients tested**	168	257	-	-
**Change in UEFM score from baseline to 2-year follow-up**				-
Total	15.14 (4.78)	2.56 (2.60)	12.58(11.88 - 13.28)	<0.0001
CONCENT-eligible	18.00 (4.86)	1.95 (1.12)	16.77(14.67 - 18.88)	<0.0001
CONCENT-ineligible	14.82 (4.67)	2.61 (2.68)	12.20(11.47 - 12.94)	<0.0001
**Secondary outcomes**				
**Number of patients tested**	168	257	-	-
**Changes in Modified Ashworth Scale score from baseline to 2-year follow-up**				
Elbow				
Total	0.88 (0.58)	0.11 (0.54)	0.77(0.66 - 0.88)	<0.0001
CONCENT-eligible	0.94 (0.66)	0.14 (0.48)	0.83(0.46 - 1.21)	0.00012
CONCENT-ineligible	0.87 (0.57)	0.11 (0.55)	0.76(0.65 - 0.88)	<0.0001
Forearm rotation				
Total	0.97 (0.73)	0.24 (0.52)	0.74(0.62 - 0.86)	<0.0001
CONCENT-eligible	1.12 (0.78)	0.19 (0.40)	0.95(0.58 - 1.31)	<0.0001
CONCENT-ineligible	0.95 (0.72)	0.24 (0.53)	0.72(0.60 - 0.84)	<0.0001
Wrist				
Total	1.10 (0.72)	0.16 (0.58)	0.94(0.82 - 1.06)	<0.0001
CONCENT-eligible	1.53 (0.80)	0.14 (0.36)	1.10(0.73 - 1.48)	<0.0001
CONCENT-ineligible	1.05 (0.70)	0.16 (0.60)	0.90(0.78 - 1.03)	<0.0001
Thumb				
Total	1.37 (0.62)	0.18 (0.46)	1.17(1.07 - 1.27)	<0.0001
CONCENT-eligible	1.59 (0.62)	0.38 (0.67)	0.88(0.41 - 1.36)	0.00090
CONCENT-ineligible	1.34 (0.62)	0.16 (0.44)	1.18(1.07 - 1.28)	<0.0001
Fingers 2-5				
Total	1.04 (0.75)	0.20 (0.59)	0.87(0.74 - 0.99)	<0.0001
CONCENT-eligible	1.12 (0.49)	0.29 (0.72)	0.86(0.55 - 1.17)	<0.0001
CONCENT-ineligible	1.03 (0.77)	0.19 (0.58)	0.86(0.73 - 0.99)	<0.0001
**Changes in Range of motion from baseline to two-year's follow-up***				
Elbow				
Total	30.54 (14.88)	-4.22 (5.73)	35.38(33.37 - 37.39)	<0.0001
CONCENT-eligible	35.00 (15.31)	-4.29 (5.31)	40.29(33.51 - 47.07)	<0.0001

CONCENT-ineligible	30.03 (14.79)	-4.21 (5.78)	34.85(32.75 - 36.95)	<0.0001
Forearm rotation				
Total	38.21 (15.94)	-2.38 (3.41)	41.47(39.44 - 43.49)	<0.0001
CONCENT-eligible	39.12 (15.64)	-2.14 (2.99)	41.80(35.03 - 48.58)	<0.0001
CONCENT-ineligible	38.11 (16.02)	-2.40 (3.45)	41.43(39.30 - 43.56)	<0.0001
Wrist				
Total	38.54 (16.13)	-2.28 (3.85)	41.47(39.39 - 43.55)	<0.0001
CONCENT-eligible	45.00 (15.61)	-2.38 (4.07)	47.06(40.41 - 53.71)	<0.0001
CONCENT-ineligible	37.81 (16.08)	-2.27 (3.84)	40.77(38.59 - 42.95)	<0.0001
**Patient report outcome ¶**				
**Number of patients tested**	154	220		
Total Satisfaction				
Total	3.98 (1.06)	1.90 (0.83)	2.08(1.89 - 2.27)	<0.0001
CONCENT-eligible	4.33 (0.62)	1.71 (0.56)	2.62(2.23 - 3.01)	<0.0001
CONCENT-ineligible	3.94 (1.10)	1.92 (0.85)	2.02(1.82 - 2.23)	<0.0001
Improvement of Self-caring ability				
Total	3.68 (1.06)	1.86 (0.80)	1.82(1.63 - 2.00)	<0.0001
CONCENT-eligible	3.93 (1.03)	1.81 (0.68)	2.12(1.57 - 2.68)	<0.0001
CONCENT-ineligible	3.65 (1.06)	1.86 (0.81)	1.78(1.59 - 1.98)	<0.0001
Reduction of Family burden of caring				
Total	3.64 (1.07)	1.85 (0.82)	1.79(1.60 - 1.97)	<0.0001
CONCENT-eligible	3.93 (1.10)	1.76 (0.70)	2.17(1.58 - 2.76)	<0.0001
CONCENT-ineligible	3.60 (1.06)	1.86 (0.83)	1.75(1.55 - 1.94)	<0.0001
Usual activities				
Total	3.90 (1.04)	1.81 (0.80)	2.09(1.91 - 2.27)	<0.0001
CONCENT-eligible	4.33 (0.82)	1.57 (0.60)	2.76(2.30 - 3.22)	<0.0001
CONCENT-ineligible	3.86 (1.05)	1.83 (0.81)	2.02(1.83 - 2.22)	<0.0001
Pain or discomfort release				
Total	3.26 (1.08)	1.81 (0.87)	1.45(1.25 - 1.65)	<0.0001
CONCENT-eligible	3.73 (1.03)	1.63 (0.68)	2.10(1.52 - 2.68)	<0.0001
CONCENT-ineligible	3.21 (1.08)	1.83 (0.89)	1.38(1.17 - 1.59)	<0.0001
Anxiety or depression release				
Total	3.25 (1.14)	1.72 (0.81)	1.53(1.33 - 1.73)	<0.0001
CONCENT-eligible	3.40 (1.35)	1.63 (0.76)	1.77(1.05 - 2.49)	<0.0001
CONCENT-ineligible	3.23 (1.12)	1.73 (0.82)	1.51(1.30 - 1.71)	<0.0001

¶ Patient report outcomes include six aspects, including total satisfactory of the treatment, change of self-caring ability, reduction of family burden of caring, increase of efficiency and ability of usual activities, release of pain or discomfort, release of anxiety or depression. Each question was analyzed independently, and scores were rated in five levels from 1 to 5, with higher scores indicating better satisfaction. Part of the data was missing in this evaluation, including 14 missing in the surgery group and 15 missing in the control group.

Table S2. Patient reported outcomes in the matched cohort.

Outcome	Mean (SD)		Mean(95%CI)	P-value
	Surgery	Rehabilitation	Adjusted Mean difference	
**Number of patients tested**	154	153		
**Total Satisfaction**				
Total	3.98 (1.06)	1.88 (0.85)	2.10(1.89 - 2.31)	<0.0001
CONCENT-eligible	4.33 (0.62)	1.62 (0.51)	2.72(2.30 - 3.14)	<0.0001
CONCENT-ineligible	3.94 (1.10)	1.90 (0.87)	2.04(1.81 - 2.26)	<0.0001
**Improvement of Self-caring ability**				
Total	3.68 (1.06)	1.86 (0.84)	1.82(1.61 - 2.03)	<0.0001
CONCENT-eligible	3.93 (1.03)	1.69 (0.63)	2.24(1.59 - 2.89)	<0.0001
CONCENT-ineligible	3.65 (1.06)	1.87 (0.85)	1.78(1.56 - 2.00)	<0.0001
**Reduction of Family burden of caring**				
Total	3.64 (1.07)	1.87 (0.86)	1.77(1.56 - 1.98)	<0.0001
CONCENT-eligible	3.93 (1.10)	1.69 (0.63)	2.24(1.56 - 2.92)	<0.0001
CONCENT-ineligible	3.60 (1.06)	1.88 (0.88)	1.72(1.50 - 1.94)	<0.0001
**Usual activities**				
Total	3.90 (1.04)	1.83 (0.83)	2.08(1.87 - 2.28)	<0.0001
CONCENT-eligible	4.33 (0.82)	1.54 (0.52)	2.79(2.28 - 3.31)	<0.0001
CONCENT-ineligible	3.86 (1.05)	1.85 (0.85)	2.00(1.79 - 2.22)	<0.0001
**Pain or discomfort release**				
Total	3.26 (1.08)	1.82 (0.93)	1.44(1.21 - 1.66)	<0.0001
CONCENT-eligible	3.73 (1.03)	1.55 (0.69)	2.19(1.48 - 2.89)	<0.0001
CONCENT-ineligible	3.21 (1.08)	1.85 (0.94)	1.37(1.13 - 1.60)	<0.0001
**Anxiety or depression release**				
Total	3.25 (1.14)	1.68 (0.82)	1.57(1.35 - 1.79)	<0.0001
CONCENT-eligible	3.40 (1.35)	1.36 (0.67)	2.04(1.16 - 2.91)	0.00012
CONCENT-ineligible	3.23 (1.12)	1.70 (0.83)	1.53(1.30 - 1.76)	<0.0001

¶ Patient report outcomes consists of six aspects, including total satisfactory of the treatment, change of self-caring ability, reduction of family burden of caring, increase of efficiency and ability of usual activities, release of pain or discomfort, release of anxiety or depression. Each question was analyzed independently, and scores were rated in five levels from 1 to 5, with higher scores indicating better satisfaction. Part of the data was missing in this evaluation, including 14 missing in the surgery group and 15 missing in the control group.

Table S3. Longitudinal data at baseline and time points of each follow-up in the surgery population

Outcome	Baseline	Month 3	Month 6	Year 1	Year 2	Year 3	Year 5
**Total UEFM score**	24.80 (12.55)	26.27 (12.09)	28.04 (12.09)	35.90 (13.64)	39.94 (13.54)	41.42 (11.11)	45.77 (7.34)
Number of patients tested	168	131	126	165	168	64	26
**Subgroups**							
Rehabilitation after surgery							
Regular Rehabilitation	24.85 (10.98)	26.51 (10.25)	28.36 (9.96)	37.32 (11.83)	41.51 (11.73)	44.10 (8.96)	46.43 (7.37)
No regular rehabilitation	24.63 (17.03)	25.39 (17.49)	26.85 (18.10)	31.00 (17.94)	34.58 (17.57)	30.92 (12.81)	40.67 (5.77)
Age [a]							
<12	28.43 (9.24)	28.86 (8.51)	30.29 (7.78)	41.14 (9.70)	47.00 (10.12)	43.40 (10.06)	48.00 (5.23)
12-45	25.93 (12.39)	28.14 (12.02)	29.54 (12.08)	37.24 (13.69)	41.07 (13.31)	42.13 (11.39)	45.45 (8.08)
>45	22.09 (12.95)	21.90 (11.83)	24.00 (12.15)	32.48 (13.47)	36.80 (13.86)	37.83 (10.48)	44.50 (0.71)
Age [b]							
<18	30.44 (14.77)	32.43 (13.71)	33.77 (14.20)	42.25 (15.72)	46.52 (15.52)	46.86 (12.31)	48.43 (7.50)
18-45	24.85 (11.17)	26.76 (10.76)	28.26 (10.66)	36.19 (12.60)	40.00 (12.16)	40.55 (10.41)	44.82 (7.67)
>45	22.09 (12.95)	21.90 (11.83)	24.00 (12.15)	32.48 (13.47)	36.80 (13.86)	37.83 (10.48)	44.50 (0.71)
Cause of injury							
Stoke							
Hemorrhagic	22.65 (11.54)	24.35 (9.21)	26.53 (10.23)	34.49 (12.38)	38.46 (12.27)	43.06 (7.46)	45.00 (1.00)
Ischemic	22.82 (13.62)	22.71 (13.55)	24.48 (13.35)	32.03 (14.66)	36.56 (14.80)	33.45 (10.38)	44.50 (0.71)
Cerebral palsy	30.78 (13.87)	32.58 (13.34)	34.08 (13.66)	44.08 (14.14)	47.56 (13.84)	46.57 (10.92)	50.62 (9.75)
Traumatic brain injury	25.78 (11.22)	26.50 (11.28)	27.43 (10.75)	36.59 (12.62)	39.97 (12.56)	41.00 (12.69)	42.56 (5.66)
Encephalitis	27.57 (9.98)	27.86 (10.93)	29.71 (9.71)	35.57 (12.16)	42.57 (12.12)	40.83 (11.16)	44.50 (6.61)
Duration of disease — y							
<5	23.00 (11.79)	24.44 (11.48)	26.24 (11.10)	34.70 (12.98)	38.96 (12.98)	40.81 (11.11)	44.60 (2.30)
⩾5	26.51 (13.07)	27.78 (12.44)	29.44 (12.71)	37.06 (14.22)	40.87 (14.05)	41.84 (11.25)	46.05 (8.12)
Severity (UEFM score at baseline)							
<20	11.59 (4.89)	12.95 (5.05)	14.32 (5.44)	21.71 (7.11)	25.12 (7.27)	28.28 (8.04)	33.67 (2.52)
20-40	28.08 (5.26)	29.21 (5.78)	31.21 (5.26)	40.79 (7.66)	45.20 (6.93)	45.57 (6.61)	47.00 (6.09)

>40	46.39 (5.30)	46.76 (4.80)	48.44 (4.70)	55.32 (5.02)	58.30 (4.02)	57.00 (5.35)	55.00 (NA)
Nerve graft or Direct coaptation							
Direct coaptation	24.34 (12.52)	25.99 (11.83)	27.63 (12.09)	35.57 (13.56)	39.54 (13.48)	41.91 (10.56)	45.47 (6.72)
Nerve graft	26.31 (12.69)	26.97 (12.84)	29.06 (12.21)	36.97 (14.02)	41.26 (13.81)	40.43 (12.38)	46.33 (8.82)
**Secondary outcomes**							
MAS---Mean (SD)							
Number of patients tested	168	131	126	165	168	64	26
Elbow	2.12 (0.79)	1.66 (0.70)	1.94 (0.73)	1.58 (0.83)	1.24 (0.86)	1.05 (0.70)	0.96 (0.72)
Forearm rotation	2.30 (0.76)	1.73 (0.74)	2.11 (0.67)	1.61 (0.90)	1.33 (0.89)	1.19 (0.85)	1.00 (0.75)
Wrist	2.42 (0.84)	1.73 (0.70)	2.24 (0.74)	1.58 (0.79)	1.32 (0.80)	1.20 (0.76)	1.04 (0.72)
Thumb	2.49 (0.88)	1.73 (0.80)	2.28 (0.82)	1.61 (0.74)	1.12 (0.78)	1.17 (0.77)	0.96 (0.66)
Fingers 2-5	2.19 (0.92)	1.52 (0.80)	2.02 (0.81)	1.36 (0.88)	1.15 (0.86)	1.06 (0.89)	0.92 (0.74)
Range of motion---degree							
Number of patients tested	168	131	126	165	168	62	26
Elbow	77.95 (30.79)	79.89 (29.15)	89.09 (27.85)	102.06 (27.46)	108.48 (26.51)	110.73 (25.20)	118.85 (17.62)
Forearm rotation	39.26 (27.25)	40.19 (21.79)	44.21 (21.82)	66.06 (25.60)	77.47 (25.76)	75.40 (22.03)	75.38 (24.00)
Wrist	54.76 (23.96)	55.27 (19.59)	58.29 (20.30)	79.91 (24.65)	93.30 (22.72)	91.05 (22.64)	97.12 (16.26)

The values in this table refers to the total score of UEFM assessments in each follow-up. The subgroups of age were divided according to two criteria: a: Subgroup of age divided according to CONCENT-eligible or CONCENT-ineligible. b. Subgroup of age divided according to adulthood.

Table S4. Extended data for changes on muscle strength in the surgery group.

Muscle strength scores—No. of patients	Score	Baseline		Month 1		Month 3		Month 6	
		Intact	Paralyzed	Intact	Paralyzed	Intact	Paralyzed	Intact	Paralyzed
**Elbow extension**	0	0	0	0	0	0	0	0	0
	1	0	0	0	0	0	0	0	0
	2	0	13	0	55	0	16	0	11
	3	0	55	40	70	8	52	0	54
	4	0	61	85	32	34	66	6	64
	5	168	39	43	11	126	34	162	39
**Wrist extension**	0	0	13	0	30	0	12	0	12
	1	0	32	0	42	0	35	0	30
	2	0	49	0	49	0	45	0	47
	3	0	42	15	34	0	41	0	42
	4	0	24	53	13	46	25	2	26
	5	168	8	100	0	122	10	166	10
**Finger extension**	0	0	20	0	35	0	18	0	17
	1	0	34	0	44	0	30	0	31
	2	0	48	0	40	0	46	0	47
	3	0	35	7	37	0	42	0	41
	4	0	25	43	12	23	26	0	26
	5	168	6	118	0	145	6	168	6

The muscle strength of elbow, wrist, and finger was evaluated at baseline and at follow-ups in accordance with the Medical Research Council grading system on a scale of 0 to 5, with higher scores indicating greater muscle power.

Table S5. Adverse events

Events	Initial adverse events (1 month)				Persistent adverse events (6 months)			
	CONCENT-eligible		CONCENT-ineligible		CONCENT-eligible		CONCENT-ineligible	
**Complications related to treatment [a] – no. of events**	Surgery group (n=17)	Rehabilitation group (n=13)	Surgery group (n=151)	Rehabilitation group (n=155)	Surgery group (n=17)	Rehabilitation group (n=13)	Surgery group (n=151)	Rehabilitation group (n=155)
**Bleeding**	0	0	0	0	0	0	0	0
**Infection**	0	0	0	0	0	0	0	0
**Pain**	7	2	91	7	0	0	1*	0
**Foreign body sensation while swallowing**	3	0	26	0	0	0	0	0
**Fatigue**	5	0	49	12	2*	0	2*	3
**Transient palsy of the phrenic nerve**	0	0	5	0	0	0	0	0
**Lymphorrhea**	1	0	3	0	0	0	0	0
**Changes in sensorimotor function – no. of events [b]**								
**Numbness**	14	0	145	0	0	0	0	0
**Decrease in muscle strength-intact side**								
**Elbow**	15	0	110	0	1	0	5	0
**Wrist**	10	0	58	0	0	0	2	0
**Fingers**	8	0	43	0	0	0	0	0
**Decrease in muscle strength-paralyzed side**								
**Elbow**	7	0	78	0	0	0	0	0
**Wrist**	5	0	31	0	0	0	0	0
**Fingers**	5	0	29	0	0	0	0	0
**Tactile sensory threshold increase in thumb, index and middle fingers [c]**	3	0	32	0	0	0	0	0

[a] The principal investigator determined whether a complication was related to the surgery or rehabilitation therapy. [b] The power of elbow, wrist, and finger was evaluated at baseline and at follow-ups in accordance with the Medical Research Council grading system on a scale of 0 to 5, with higher scores indicating greater muscle power. [c] Tactile sensory threshold is the weakest stimulus that an organism can detect. The tactile sensory threshold was measured in both thumbs, index fingers, and middle fingers with Semmes–Weinstein monofilaments. * The pain and fatigue sustained for 6 months and disappeared at year 1.

Table S6. Sensitivity analysis on primary outcome.

	Difference|| (Mean, 95% CI)	P-value
**Multivariate model†**		
Total population	12.61 (11.86-13.35)	<0.0001
Chinese population	13.19 (12.48-13.89)	<0.0001
**Propensity-score weighting model‡**	12.59 (11.89-13.29)	<0.0001

||The difference of changes on total score of UEFM scale from baseline to 2-year follow-up between surgery and control group. In multivariate model† of total population and Chinese population, the following factors were all included as covariates: age, sex, education level, body-mass index, smoking, comorbidity including diabetes mellitus and hypertension, duration of disease, score of UEFM scale at baseline, cause of injury, paralyzed side and center. In multivariate model of Chinese patients, patients from four center in China were included. In propensity-score weighting model‡, the covariates were same with multivariate model, and a total of 425 patients were included.

Figure S1. Subgroup analyses of the primary outcome in the surgery group.

Subgroups	No. of Patients	Mean Change from baseline to two-year's follow-up
Total	168	15.14(14.42~15.87)
Population		
CONCENT−eligible	***17***	***18.00(15.50~20.50)***
CONCENT−ineligible	151	14.82(14.07~15.57)
Age−yr [a]		
<12	7	18.57(14.65~22.49)
12−45	***107***	***15.14(14.20~16.08)***
>45	54	14.70(13.47~15.94)
Age−yr [b]		
<18	25	16.08(13.84~18.32)
18−45	89	15.15(14.14~16.15)
>45	54	14.70(13.47~15.94)
Gender		
Female	***26***	***14.35(12.00~16.70)***
Male	142	15.29(14.53~16.05)
Education		
Junior high school or below	44	15.36(13.84~16.89)
High school and above	124	15.06(14.23~15.90)
Baseline UEFM score		
<20	59	13.53(12.53~14.52)
20−40	***86***	***17.12(16.08~18.16)***
>40	23	11.91(10.49~13.33)
Duration of Disease— yr		
<5	82	15.96(15.03~16.89)
≥5	***86***	***14.36(13.26~15.46)***
Cause of injury		
Stoke		
Hemorrhagic	63	15.81(14.71~16.90)
Ischemic	39	13.74(12.21~15.28)
Cerebral palsy	27	16.78(14.42~19.13)
Traumatic brain injury	32	14.19(12.71~15.67)
Encephalitis	7	15.00(10.66~19.34)
Side of paralyzed hand		
Left	94	15.06(14.08~16.05)
Right	74	15.24(14.13~16.35)
Nerve graft or Direct coaptation		
Direct anastomosis	***129***	***15.20(14.39~16.02)***
Nerve graft	39	14.95(13.28~16.62)
Rehabilitation − mon		
<6	38	9.95(9.03~10.86)
≥6	***130***	***16.66(15.95~17.38)***
Center		
Huashan hospital	***132***	***15.43(14.58~16.28)***
Other centers	36	14.08(12.75~15.42)

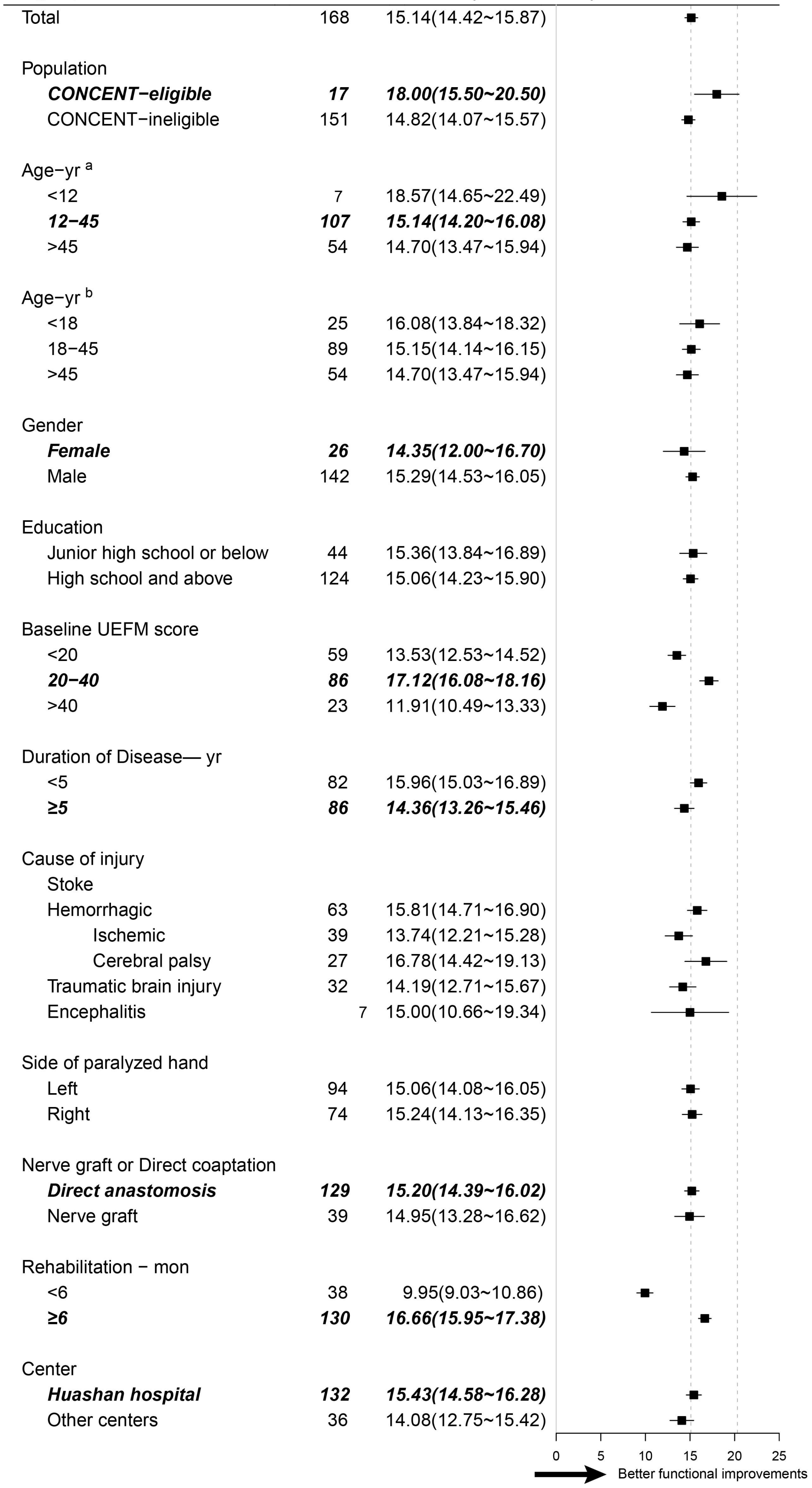

Italic Bold style represented subgroups in accordant with the CONCENT criteria. Areas between dotted lines showed the confident intervals of the differences of the changes in UEFM. Areas between dotted lines showed the confident intervals of the differences of the changes in UEFM score from baseline to two-year's follow-up. a: Subgroup of age divided according to CONCENT-eligible or CONCENT-ineligible. b. Subgroup of age divided according to adulthood.

Figure S2. Patient report outcomes on the reason of why patients in the control group choose rehabilitation over surgery.

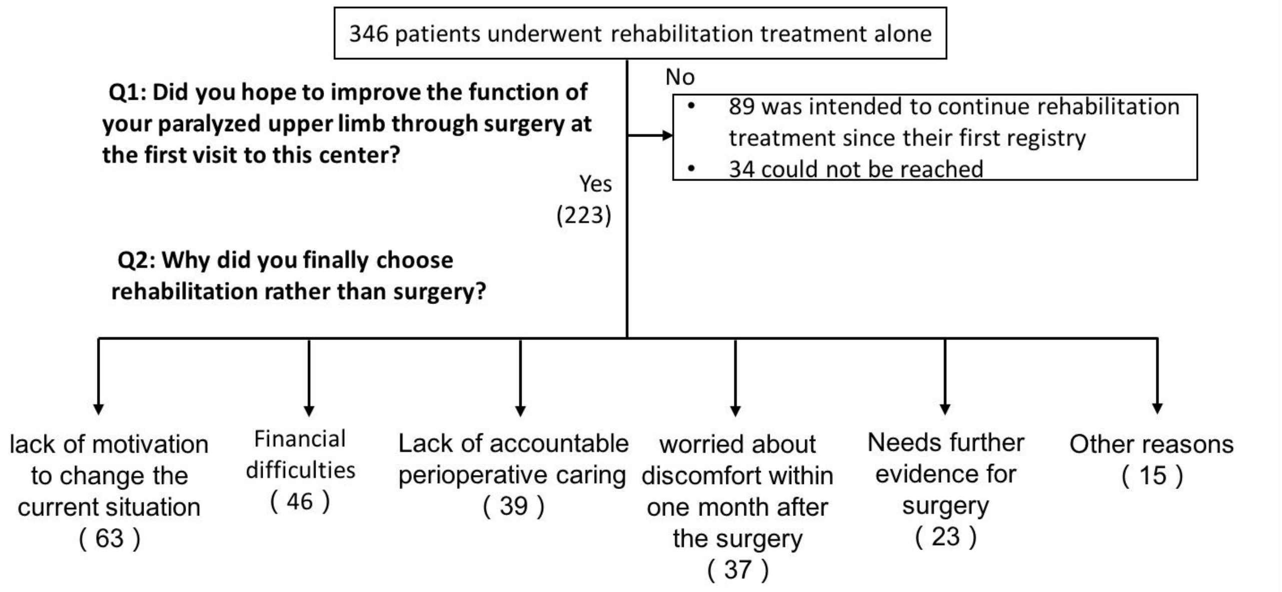

This questionnaire is consisted of two questions: a. did you hope to improve your paralyzed upper limb function through surgery at your first visit to this center? b. Why did you finally choose rehabilitation rather than surgery? For the second question, the answers included financial difficulties, lack of accountable perioperative caring, worried about discomfort within one month after the surgery, lack of motivation to change the current situation, still needs to wait and see further reports of the surgery, worried about complications of the surgery, and an open option for other reasons. Patients should choose one of the options to be the main reason, and free to choose more options as other reasons.

Figure S3. Extended data for adverse events in muscle strength of the surgery group.

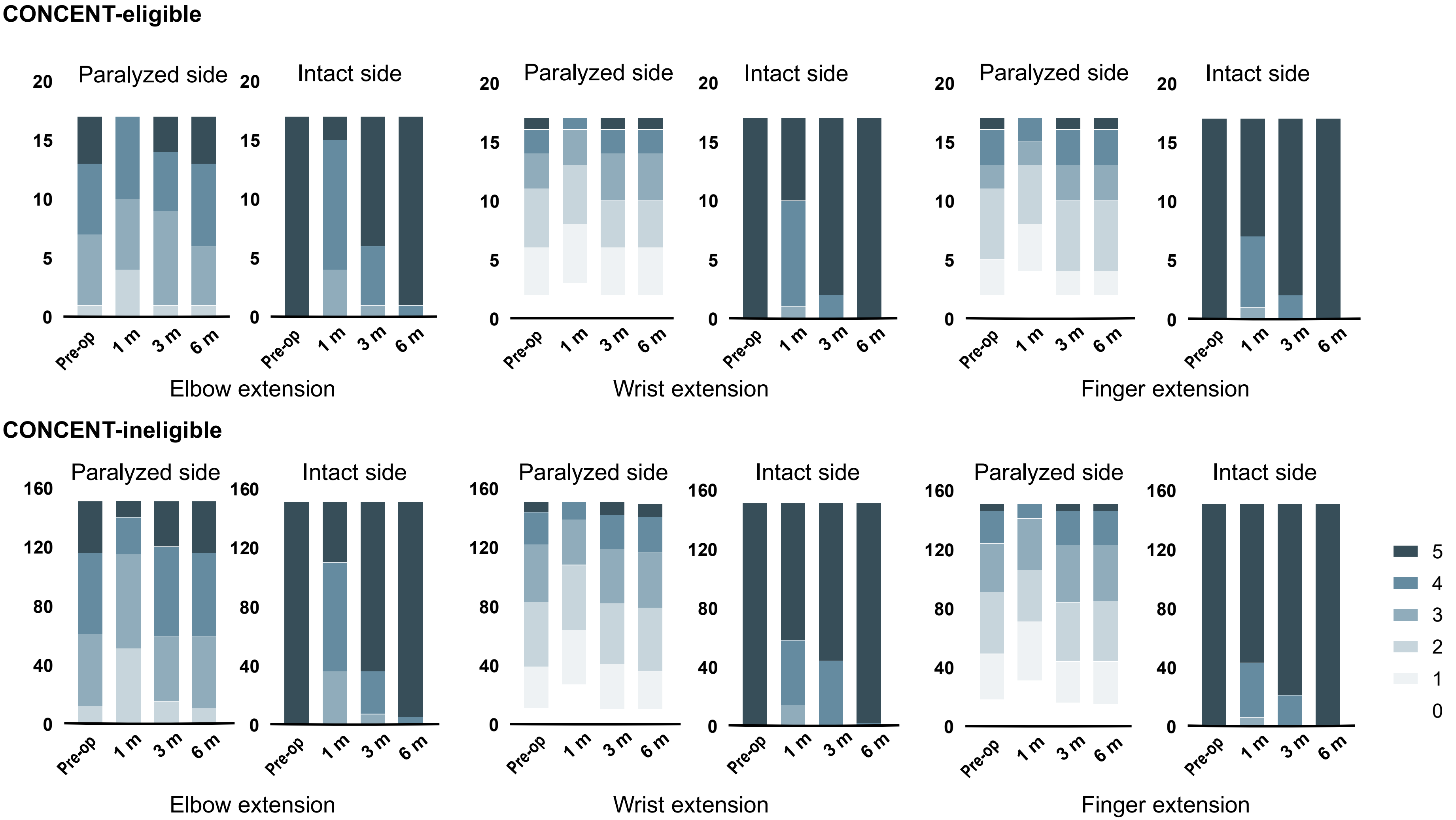

The frequency chart of patient number in each muscle strength level in elbow extension, wrist extension, and finger extension. The muscle strength is evaluated in accordance with the Medical Research Council grading system on a scale of 0 to 5.

Figure S4. Longitudinal data of MAS score in the surgery group.

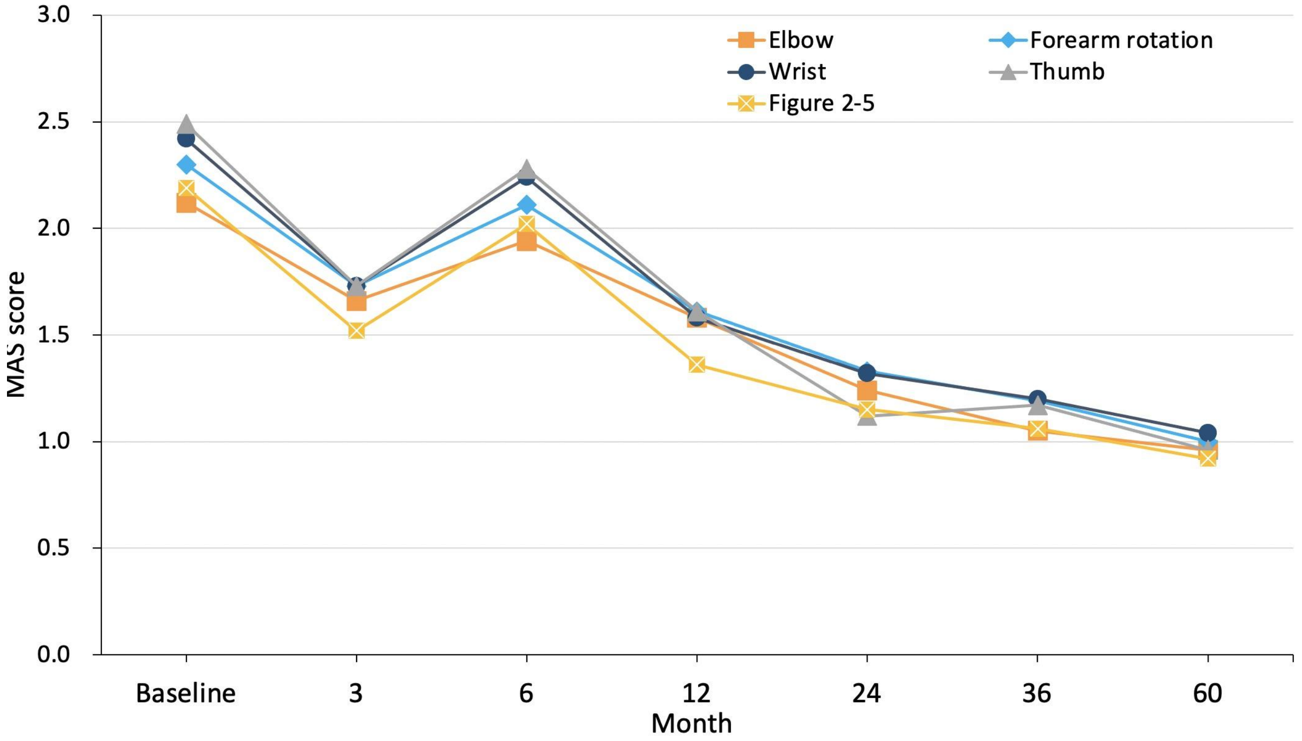

The dots in this chart indicated the mean value of MAS score of each joint at each follow-up point. The MAS score indicated the spasticity of the evaluated joint, with a range of 0 to 5, and higher MAS score indicates higher spasticity.

Supporting information 1. Description of the treatments in the surgery and rehabilitation group.

The contralateral cervical seventh nerve transfer surgery is performed in the following steps: A 15-cm transverse incision is made approximately 2 cm superior to the clavicle at the bottom of the neck. Bilateral brachial plexus nerves are exposed at the level of supraclavicular triangle. On the intact side, the C7 nerve on the paralyzed side is severed near the intervertebral foramen, and the C7 nerve on the nonparalyzed side is severed as distally as possible, proximal to the point at which it combines with the fibers of other brachial plexus nerves. The anterolateral aspect of the C7 vertebral body is dissected bluntly, and the esophagus is exposed anterior to the vertebral body, which creates a conduit between the spinal column and the esophagus. The cut end of the C7 nerve on the nonparalyzed side is then drawn through the prespinal route to the paralyzed side and coapted with the cut end of the C7 nerve on the paralyzed side by means of microsurgical epineurium suturing. After surgery, the paralyzed upper extremity is immobilized with a head–arm brace for 4 weeks, after which the patients have the same rehabilitation therapy as they did before the surgery.

Rehabilitation started after removing of the immobilizing cast in the surgery group. The items included active exercise, passive range of motion, occupational therapy, functional training, physical therapy, acupuncture, and usage of orthoses. In the rehabilitation group, the treatments should last for at least six months within one-year after initial assessments. In the surgery group, rehabilitation was recommended for each patient but not forced. The duration of post-operative rehabilitation was recorded.
